# Functional CRISPR Screens Define Genetic Drivers for Cancer Transformation and Progression from Non-Cancerous Cells

**DOI:** 10.3390/ijms27073223

**Published:** 2026-04-02

**Authors:** Shixin Ma, You Li, Teng Fei

**Affiliations:** 1 Key Laboratory of Bioresource Research and Development of Liaoning Province, College of Life and Health Sciences, Northeastern University, Shenyang 110819, China; mashixin0902@163.com (S.M.); nanjiliyou@163.com (Y.L.); 2 Foshan Graduate School of Innovation, Northeastern University, Foshan 528311, China; 3 National Frontiers Science Center for Industrial Intelligence and Systems Optimization, Northeastern University, Shenyang 110819, China; 4 Key Laboratory of Data Analytics and Optimization for Smart Industry (Northeastern University), Ministry of Education, Shenyang 110819, China

**Keywords:** CRISPR screen, fibroblast, immortalization, transformation, liver cancer, breast cancer, metastasis

## Abstract

Tumor initiation and metastatic progression are driven by context-dependent genetic alterations that disrupt tumor suppressor pathways, metabolic homeostasis, and signaling networks. However, the initial drivers that transform normal cells into malignant ones and their context dependency remain elusive. To address this, we aimed to systematically identify and characterize these drivers across cancer types, species, and microenvironments. We constructed customized clustered regularly interspaced short palindromic repeats (CRISPR)/CRISPR-associated protein 9 (Cas9) knockout (KO) libraries targeting high-frequency mutated and downregulated genes associated with liver hepatocellular carcinoma (LIHC) and breast carcinoma (BRCA) and conducted parallel functional screens in non-cancerous mouse and human fibroblast cell lines under two-dimensional (2D), three-dimensional (3D), and in vivo conditions. Strikingly, *TP53* and *NF1* emerged as pan-context drivers consistently enriched across immortalization, tumorigenesis, and metastasis in both LIHC and BRCA settings, while most other identified drivers were largely species-, tissue-, and microenvironment-specific with limited cross-model overlap. Despite this heterogeneity, all drivers converge on core pathways including epigenetic regulation, metabolic reprogramming, and growth factor signaling. Unlike prior studies on established cancer cells, this work defines the genetic barriers restricting the malignant transformation of primary normal cells, offering a new framework for early cancer evolution.

## 1. Introduction

Cancer development is a multistep process driven by the sequential acquisition of genetic and epigenetic alterations that collectively enable cells to bypass normal growth constraints, evade tumor suppressor surveillance, and ultimately achieve malignant transformation [1]. However, fundamental and unresolved questions remains: which specific genetic drivers initiate this transformation from non-cancerous primary cells, and how do these drivers differ across cancer types, species, and microenvironments? Addressing this gap is essential for understanding the earliest events in tumor evolution. Among the earliest and most critical steps in this process is cellular immortalization—the escape from replicative senescence—which is primarily governed by telomere attrition and the activation of the p53 or retinoblastoma (RB) tumor suppressor pathways [2,3,4]. Disruption of these checkpoints not only permits unlimited proliferative capacity but also creates a permissive cellular environment for the accumulation of additional oncogenic lesions [4].

Mouse embryonic fibroblasts (MEFs) have long served as a cornerstone model for dissecting the molecular mechanisms underlying oncogenic transformation. Under defined culture conditions, MEFs undergo spontaneous immortalization through loss-of-function alterations in p53 and/or p19^ARF^, and are highly susceptible to transformation by activated oncogenes such as RAS [5,6]. The tractability of MEFs has enabled systematic investigation of cell cycle checkpoint defects, DNA damage response dysregulation, and chromatin remodeling events that collectively constitute early tumorigenic events [7,8]. However, the relative ease with which MEFs undergo transformation contrasts sharply with the behavior of primary human fibroblasts, which exhibit substantially greater resistance to spontaneous immortalization and require defined genetic perturbations—including human telomerase reverse transcriptase (*hTERT*) reactivation, SV40 Large T antigen-mediated inactivation of p53 and RB, and oncogenic RAS activation—to acquire full tumorigenic competence [9,10]. This species-specific difference in transformation barriers underscores the importance of employing human cellular models to faithfully recapitulate the genetic requirements for human tumor initiation and progression [11].

Immortalized human fibroblast cell lines provide a valuable and experimentally tractable platform for investigating the molecular determinants of tumor growth and metastatic dissemination within a human genetic context. Once key tumor suppressor barriers are dismantled, these cells can form subcutaneous tumors in immunocompromised mice and, under appropriate selective pressures, give rise to distant metastases in vivo [9,12]. Comparative analyses between MEFs and human immortalized fibroblasts therefore offer a powerful strategy to distinguish conserved, species-independent drivers of malignant transformation from species-specific mechanisms.

Large-scale genomic profiling studies, including those conducted by The Cancer Genome Atlas (TCGA), have comprehensively characterized the mutational landscapes of liver hepatocellular carcinoma (LIHC) and breast carcinoma (BRCA), revealing recurrent high-frequency alterations in genes regulating cell cycle progression, growth factor signaling, epigenetic modification, and cellular metabolism [13,14,15,16]. Notably, a substantial subset of these genes exhibits consistent downregulation in primary tumors and metastatic lesions, implicating loss-of-function events as selective drivers of tumor evolution [14,16]. Despite these advances, the functional consequences of many such mutations remain poorly characterized, especially which of these alterations are genuine initiating events capable of driving transformation from normal, non-cancerous cells, as opposed to passenger events or late-stage adaptations.

The advent of pooled clustered regularly interspaced short palindromic repeats (CRISPR)/ CRISPR-associated protein 9 (Cas9) loss-of-function screening has revolutionized the systematic functional interrogation of cancer-relevant genes at the genome scale [17,18]. In particular, in vivo CRISPR screens have proven instrumental in identifying genes whose loss promotes tumor growth, survival, and metastatic colonization under physiologically relevant selective pressures [19,20]. However, prior CRISPR screens have been predominantly conducted in established cancer cell lines, which harbor pre-existing oncogenic backgrounds that obscure the identification of true initiating drivers. To address this critical limitation, we designed customized CRISPR/Cas9 knockout (KO) libraries targeting high-frequency mutated genes and downregulated genes in LIHC and BRCA, and performed parallel functional screens in non-cancerous MEFs and human immortalized fibroblasts through in vitro proliferation assays and in vivo tumor and metastasis models. This design allows systematic identification of the genetic barriers restricting malignant transformation of normal cells, and enables direct cross-species and cross-context comparison of driver genes. Our screens identified *TP53* and *NF1* as pan-context drivers consistently enriched across immortalization, tumorigenesis, and metastasis in both cancer settings, while revealing that most other drivers are highly context-specific. This study provides important insights into the key genetic events that drive malignant transformation and metastatic competence from normal cells across species and tumor origins.

## 2. Results

### 2.1. Construction and Validation of the mLIHC-KO Screening Platform in MEFs

**Figure 1:**
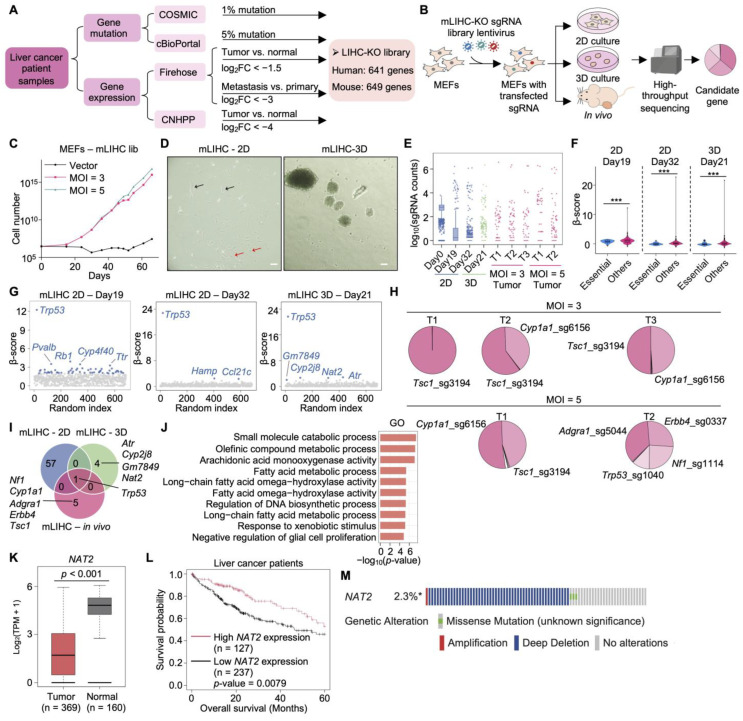
LIHC-KO screening identifies genetic drivers of MEF immortalization in 2D and 3D culture and in vivo tumorigenesis: (**A**) Design and composition of the LIHC-KO library for functional screening. (**B**) Schematic overview of the experimental workflow. MEFs were infected with the pooled mLIHC-KO library and subjected to 2D culture, 3D spheroid formation, and in vivo subcutaneous tumor formation assays for functional screening. (**C**) Cell growth assay quantifying cell numbers during 2D screening after infection with the mLIHC-KO library at multiplicities of infection (MOI) of 3 and 5. Cells transduced with empty vector were used as controls. (**D**) Representative bright-field images of MEFs subjected to 2D and 3D screening. In the 2D image, black arrows denote non-transformed MEFs, while red arrows indicate transformed MEFs. Scale bar, 200 μm. (**E**) Boxplot showing the distribution of log_10_-transformed sgRNA counts across different screening conditions. The box denotes the interquartile range (IQR), with the median indicated by a horizontal line. Whiskers represent values within 1.5 × IQR, and points outside this range are plotted as outliers. (**F**) Violin plot showing β-scores of common essential genes and others in 2D and 3D screening groups. Two-sided Wilcoxon rank-sum test, *** *p* < 0.001. (**G**) Scatter plots of β-scores from 2D and 3D screening. Genes with a β-score > 2 were considered as functional gene hits. (**H**) Pie charts showing the distribution of sgRNAs within individual subcutaneous tumors derived from in vivo screening. (**I**) Venn diagram illustrating overlapping genes identified from 2D, 3D, and in vivo tumorigenesis screening. (**J**) Heatmap showing Gene Ontology (GO) enrichment analysis of genes in (**I**). Significantly enriched biological processes are shown, ranked by *p*-value. (**K**) Boxplot showing the expression levels of *NAT2* in LIHC and normal liver tissues. The center line of each box denotes the median expression level; box boundaries indicate the 25th and 75th percentiles. Numbers in parentheses indicate sample sizes. Statistical significance was assessed using one-way ANOVA. (**L**) Kaplan–Meier analysis of overall survival in LIHC patients based on *NAT2* expression level (high expression, top one-third; low expression, lower two-thirds). Statistical significance was determined using the log-rank test. (**M**) The mutational frequency and type of *NAT2* in LIHC patients were retrieved from the cBioPortal database. * indicates that not all samples were profiled.

To systematically identify genetic drivers of MEF immortalization and tumorigenesis, we constructed a CRISPR/Cas9-based pooled knockout library targeting LIHC-associated genes (Figure 1A) [17,18]. The human LIHC-KO (hLIHC-KO) library was designed to target 641 human candidate genes selected on the basis of somatic mutation frequency, transcriptomic alterations, and proteomic downregulation identified across multiple LIHC datasets, including COSMIC [21], cBioPortal [22,23], Firehose [24], and the Chinese National Human Proteome Project (CNHPP) [25]. Following ortholog mapping, 649 murine genes were incorporated into the mouse LIHC-specific KO (mLIHC-KO) library for functional screening. In total, 4145 single guide RNAs (sgRNAs) were included in the hLIHC-KO library and 4394 sgRNAs in the mLIHC-KO library, supplemented with non-targeting controls, *AAVS1*-targeting controls, and sgRNAs directed against common essential genes (Table S1). MEFs were transduced with the pooled mLIHC-KO lentiviral library, expanded in two-dimensional (2D) monolayer culture to achieve adequate cell numbers, and subsequently divided into three parallel arms: 2D culture, three-dimensional (3D) spheroid culture [26], and in vivo subcutaneous tumorigenesis assays in mice (Figure 1B).

A prior validation of the screening platform is that transduction of the mLIHC-KO library conferred a significant proliferative advantage to primary MEFs compared to empty vector controls (Figure 1C), indicating that the ablation of LIHC-associated genes is functionally sufficient to overcome the replicative constraints of primary cells. This is further supported by the morphological transformation observed in a subset of transduced MEFs in 2D culture, and by the formation of heterogeneous spheroids in 3D methylcellulose culture—a hallmark of anchorage-independent growth capacity (Figure 1D). Critically, screening fidelity was confirmed by the robust depletion of sgRNAs targeting essential genes relative to non-essential controls across both 2D and 3D datasets (Figure 1E,F; Table S2), validating the sensitivity and specificity of the platform [27].

### 2.2. Context-Dependent Selection Identifies Trp53 as a Dominant Driver of MEF Immortalization and Tumorigenesis

A central finding of the screen is that *Trp53* inactivation is the sole genetic event consistently enriched across all three selective contexts (2D culture, 3D spheroid growth, and in vivo tumorigenesis), establishing it as a convergent and dominant driver of MEF transformation irrespective of microenvironmental conditions (Figure 1G–I). This result is biologically significant: it demonstrates that *Trp53* loss alone is sufficient to overcome the primary barriers to both cellular immortalization and tumor initiation, consistent with its well-established role as a guardian of genomic stability [28,29].

Beyond *Trp53*, context-specific hits revealed that distinct gene sets govern tumorigenesis under different conditions, pointing to the existence of multiple alternative oncogenic routes. In vivo tumor analysis revealed pronounced clonal dominance by sgRNAs targeting *Tsc1* and *Cyp1a1*, while in vitro transformed tumor cells harbored alterations in *Rb1* and *NAT2* (Figure 1G–I). The divergence between in vitro and in vivo hits highlights the importance of microenvironmental context in shaping genetic selection, and cautions against over-reliance on just 2D culture-based screens for identifying clinically relevant drivers.

Gene Ontology (GO) enrichment analysis of the candidate gene set revealed significant enrichment in metabolic processes (fatty acid metabolism, xenobiotic response, and arachidonic acid monooxygenase activity), indicating that metabolic reprogramming and detoxification pathway dysregulation contribute to MEF transformation alongside canonical tumor suppressor inactivation (Figure 1J). This finding aligns with emerging evidence that metabolic adaptation is an early and functionally important feature of oncogenic transformation.

Among the metabolic hits, *Nat2* stands out as a particularly compelling candidate driver of hepatic tumorigenesis (Figure 1I). *NAT2* encodes N-acetyltransferase 2, an enzyme that functions to both activate and deactivate arylamine and hydrazine drugs or carcinogens [30]. While *NAT2* polymorphisms have been associated with altered cancer susceptibility in colorectal and bladder cancers through modulation of carcinogen bioactivation [31], its tumor-suppressive role in hepatocellular carcinoma remains largely unexplored. The identification of *Nat2* as a hit in the 3D screen suggests that *Nat2* loss confers a selective proliferative advantage under conditions that recapitulate key features of the tumor microenvironment. This functional observation is substantiated by converging clinical evidence: *NAT2* is significantly downregulated in LIHC tumor tissues relative to normal liver (Figure 1K); reduced *NAT2* expression correlates with significantly poorer overall survival in LIHC patients (Figure 1L); and genomic alterations in LIHC are predominantly deep deletions and missense mutations (Figure 1M), consistent with its loss-of-function as the predominant mechanism of gene inactivation and tumorigenesis driver.

Taken together, these data demonstrate that LIHC-associated CRISPR/Cas9 loss-of-function screening in MEFs effectively captures both dominant and context-dependent genetic drivers of tumorigenesis. The convergence of *Trp53* across all conditions establishes it as a near-universal requirement for MEF transformation, while context-specific hits—including the metabolic regulator *Nat2*—reveal novel tumor-suppressive mechanisms with direct clinical relevance in liver cancer.

### 2.3. In Vivo hLIHC-KO Screening Identifies Tumorigenesis- and Metastasis-Promoting Genes in Human Fibroblasts

**Figure 2:**
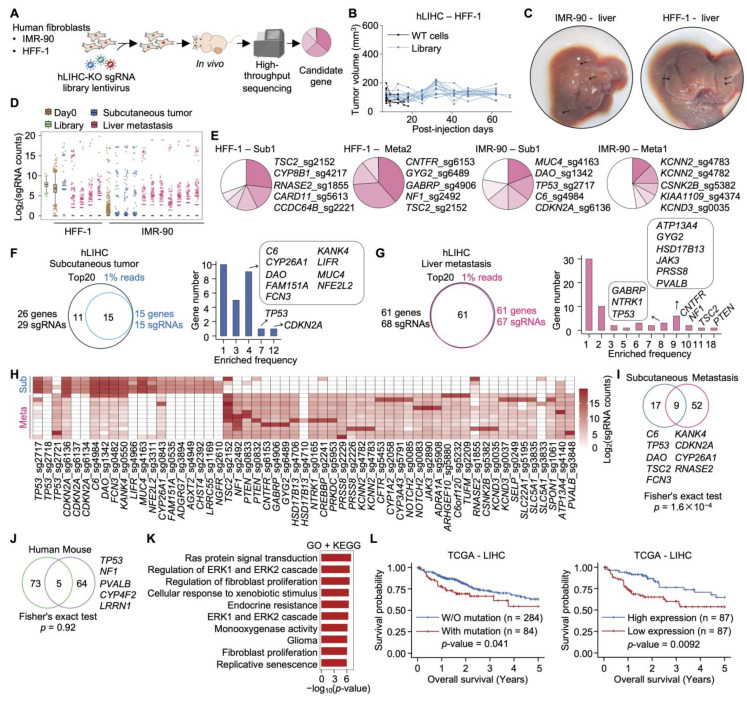
Identification of genes promoting tumorigenesis and metastasis in human fibroblast cell lines using the hLIHC-KO library: (**A**) Schematic overview of the in vivo screening workflow. Human immortalized fibroblast cells were infected with the hLIHC-KO library and injected subcutaneously into mice for tumor formation and metastasis analysis. (**B**) Tumor growth curves representing the size of subcutaneous tumors formed by HFF-1 cells in mice over time. (**C**) Representative images of livers from mice bearing IMR-90 or HFF-1 tumors with metastatic lesions. Black arrows indicate liver metastases. (**D**) Boxplot showing the distribution of log_2_-transformed sgRNA counts across the indicated groups. (**E**) Representative pie charts showing the top five enriched sgRNAs in each tumor. Groups include subcutaneous tumors and liver metastases from IMR-90 and HFF-1 cells. (**F**) **Left**: Venn diagram showing the overlap of genes represented by the top 20 sgRNAs from each subcutaneous tumor and genes corresponding to sgRNAs occupying > 1% of total reads in the same group. *Right*: Bar plot depicting the occurrence frequency of these genes across subcutaneous tumors, with genes labeled. (**G**) **Left**: Venn diagram showing the overlap of genes represented by the top 20 sgRNAs from each liver metastasis and genes corresponding to sgRNAs occupying > 1% of total reads in the same group. *Right*: Bar plot illustrating the occurrence frequency of these genes across liver metastases, with genes indicated. (**H**) Heatmap of log_2_-transformed sgRNA counts for sgRNAs that appeared in the top 20 sgRNA list of at least two tumors in total across subcutaneous tumors and liver metastases. (**I**) Venn diagram showing overlap of genes corresponding to the top 20 sgRNAs in subcutaneous tumors and liver metastases. (**J**) Comparison of human fibroblast-derived tumorigenesis- and metastasis-related genes with MEF immortalization- and tumorigenesis-related genes. The Venn diagram illustrates the shared genes. (**K**) Heatmap showing GO and Kyoto Encyclopedia of Genes and Genomes (KEGG) enrichment analysis of genes identified in human fibroblasts as promoting tumorigenesis and metastasis. (**L**) Kaplan–Meier analysis of overall survival in TCGA-LIHC patients based on tumorigenesis- and metastasis-related genes. **Left**: Survival stratified by mutational status. *Right*: Survival stratified by gene expression levels (high expression, top quartile; low expression, bottom quartile). Statistical significance was determined using the log-rank test.

To determine whether loss of LIHC-associated genes promotes tumorigenesis and metastatic dissemination in human cells, immortalized human fibroblast cell lines (HFF-1 and IMR-90) were transduced with the pooled hLIHC-KO library and subjected to in vivo subcutaneous xenograft assays in nude mice (Figure 2A). Subcutaneous tumors and hepatic metastatic lesions were harvested at experimental endpoints for sgRNA sequencing and downstream analysis (Table S3).

In HFF-1 cells, wild-type (WT) control cells failed to sustain tumor growth, with no measurable tumors detected within 20 days post-injection. In contrast, hLIHC-KO-transduced HFF-1 cells formed persistent subcutaneous tumors that did not regress over the observation period (Figure 2B). Consistent with this, library-transduced IMR-90 cells generated measurable tumors (Figure S1A), collectively indicating that loss of specific LIHC-associated genes enables the tumorigenic conversion of otherwise non-tumorigenic human fibroblasts.

At the experimental endpoint, macroscopically visible hepatic metastatic nodules were identified in mice bearing tumors derived from both HFF-1 and IMR-90 library-transduced cells (Figure 2C), demonstrating that knockout of certain genes not only facilitates primary tumor formation but also confers metastatic competence.

Analysis of sgRNA distributions revealed that in vivo tumor evolution imposes strong and progressive selective pressure, driving clonal expansion of cells harboring specific gene knockouts (Figure 2D). Critically, hepatic metastases displayed the most pronounced clonal skewing in both HFF-1 and IMR-90 cells, suggesting that metastatic colonization requires a more stringent genetic selection than primary tumor growth alone.

Importantly, sgRNA enrichment profiles diverged substantially between primary subcutaneous tumors and matched hepatic metastases (Figure 2E and Figure S1B), revealing genetically distinct determinants of local tumor growth versus metastatic dissemination. This dichotomy carries significant biological implications: the genetic prerequisites for traversing the metastatic cascade—encompassing intravasation, hematogenous dissemination, and hepatic colonization—are only partially shared with those sufficient to sustain primary tumor establishment, indicating that metastatic progression is governed by a discrete and more stringent set of genetic dependencies.

### 2.4. Distinct but Partially Overlapping Gene Sets Drive Primary Tumor Growth and Liver Metastasis

To systematically identify recurrently enriched drivers, we analyzed genes represented among the top 20 sgRNAs per tumor and those accounting for more than 1% of total sequencing reads per sample. In subcutaneous tumors, recurrently enriched genes included the well-established tumor suppressors *CDKN2A* and *TP53*, along with *C6*, *DAO*, *FCN3*, *KANK4*, *LIFR*, *MUC4*, and *NFE2L2* (Figure 2F). In hepatic metastases, recurrently identified genes included *PTEN*, *TSC2*, *NF1*, and *CNTFR* (Figure 2G). With the exception of *CNTFR*, these genes represent classical tumor suppressors, underscoring the central importance of tumor suppressor pathway inactivation in driving metastatic progression, and suggests that the PI3K–mTOR and RAS–MAPK axes represent critical vulnerabilities at this stage of disease. Heatmap analysis confirmed robust and reproducible enrichment of these sgRNAs across multiple independent tumor samples (Figure 2H).

Comparative analysis identified 26 enriched genes in subcutaneous tumors and 61 genes in hepatic metastases, with nine genes overlapping between the two groups (Figure 2I). This partial overlap indicates that despite certain gene losses contribute to both primary tumor growth and metastatic dissemination, additional context-specific driver events are still required for successful metastatic colonization.

Cross-species comparison of human fibroblast-derived hits with gene hits identified in the MEF-based mLIHC-KO screening revealed 78 human candidate genes and 69 murine candidate genes, with only five genes shared between the two species (Figure 2J). This limited overlap likely reflects meaningfully biological differences between species. Human and murine cells differ fundamentally in key determinants of cellular transformation, including telomere length maintenance, the stringency of oncogene-induced senescence responses, and the basal activity of tumor suppressor networks, parameters collectively known to modulate the genetic threshold at which a given gene becomes rate-limiting for malignant transformation. Furthermore, species-specific differences in the regulatory architecture of LIHC-associated gene networks may mean that orthologous pathways are engaged through distinct genetic nodes across species. The possibility that differences in experimental systems (cell type, transduction efficiency, and selective pressure) contribute to this divergence cannot be excluded. However, the fact that canonical tumor suppressors (*TP53*, *CDKN2A*, *PTEN*) were identified in the human screen but not universally in the murine system suggests that transformation thresholds and genetic dependencies are genuinely species-context dependent. These findings highlight the complementary value of parallel cross-species screening and caution against direct extrapolation of murine genetic dependencies to human disease.

### 2.5. Functional Enrichment and Clinical Relevance of Human Tumorigenesis- and Metastasis-Related Genes

GO and Kyoto Encyclopedia of Genes and Genomes (KEGG) enrichment analysis of human fibroblast-derived candidate genes revealed significant enrichment including Ras protein signal transduction, the regulation of the ERK1/ERK2 cascade, the regulation of fibroblast proliferation, cellular response to xenobiotic stimulus, endocrine resistance, monooxygenase activity, and replicative senescence (Figure 2K). The enrichment of Ras/ERK signaling-related terms suggests that disruption of negative regulatory nodes within this pathway facilitates aberrant oncogenic signaling and tumor progression. The RAS/RAF/MEK/ERK cascade represents one of the most frequently dysregulated pathways in human cancers, with loss of upstream negative regulators such as NF1 and TSC2 leading to constitutive pathway activation and uncontrolled cell proliferation. Concurrent enrichment of xenobiotic metabolism and oxidoreductase-related processes suggests potential metabolic reprogramming in the tumor microenvironment. These pathways, which include cytochrome P450-mediated detoxification and redox regulation, have been implicated in cancer cell adaptation to oxidative stress and therapeutic resistance [32,33,34]. Additionally, the enrichment of terms related to replicative senescence and fibroblast proliferation regulation underscores the critical role of senescence bypassing mechanisms in enabling unlimited replicative potential, a fundamental hallmark of cancer [4,35].

The clinical relevance of these candidate genes was assessed using TCGA-LIHC data (Figure 2L). Patients harboring somatic mutations in tumorigenesis- and metastasis-related genes exhibited significantly poorer overall survival compared with patients without such alterations. Consistently, patients with low expression of these genes demonstrated worse five-year overall survival relative to those with high expression. These findings are consistent with prior reports linking tumor suppressor inactivation and metabolic dysregulation to adverse clinical outcomes in hepatocellular carcinoma.

Collectively, these results demonstrate that CRISPR-based in vivo screening with the hLIHC-KO library identifies distinct but partially overlapping gene sets that promote primary tumor growth and hepatic metastasis. Many of these driver genes function as tumor suppressors and are clinically associated with poor prognosis in LIHC patients, highlighting their potential relevance to liver cancer progression and metastatic disease.

### 2.6. mBRCA-KO Screening Promotes MEF Immortalization in 2D and 3D Culture

**Figure 3:**
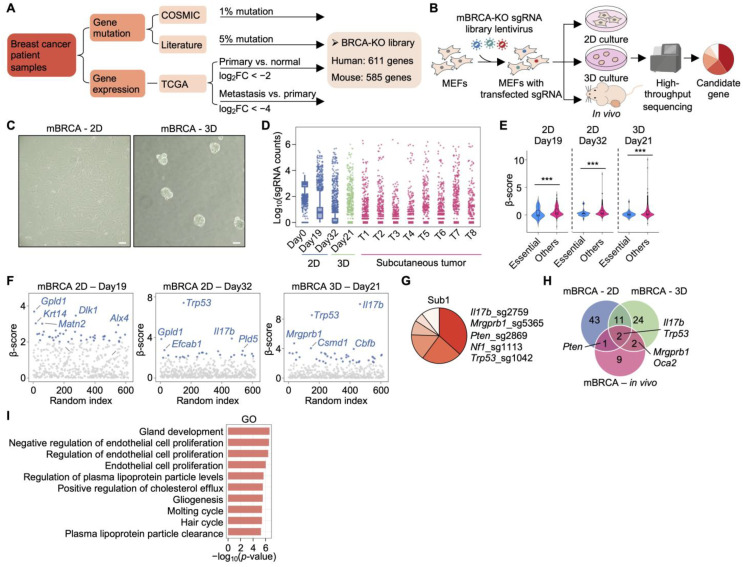
BRCA-KO screening identifies genetic drivers of MEFs immortalization in 2D and 3D culture and in vivo tumorigenesis: (**A**) Design and composition of the BRCA-KO library for functional gene hits screening. (**B**) Schematic overview of the experimental workflow. MEFs were infected with the pooled mBRCA-KO library and subjected to 2D culture, 3D spheroid formation, and in vivo subcutaneous tumor formation assays for functional screening. (**C**) Representative bright-field images of MEFs subjected to 2D and 3D screening. Scale bar, 200 μm. (**D**) Boxplot showing the distribution of log_10_-transformed sgRNA counts across different screening conditions. (**E**) Violin plot showing β-scores of common essential genes and others in 2D and 3D screening groups. Two-sided Wilcoxon rank-sum test, *** *p* < 0.001. (**F**) Scatter plots of β-scores from 2D and 3D screening. Genes with a β-score > 2 were considered as functional gene hits. (**G**) Pie chart showing the distribution of top five most enriched sgRNAs in a subcutaneous tumor derived from in vivo screening. (**H**) Venn diagram illustrating overlapping genes identified from 2D, 3D, and in vivo tumorigenesis screening. (**I**) Heatmap showing GO enrichment analysis of genes in (**H**). Significantly enriched biological processes are shown, ranked by *p*-value.

To investigate whether loss of breast cancer-associated genes promotes cellular transformation, we performed parallel in vitro and in vivo CRISPR/Cas9 loss-of-function screening using a BRCA-specific KO library (BRCA-KO) (Figure 3A) [36]. MEFs transduced with the pooled mouse BRCA-KO (mBRCA-KO) library were expanded in 2D culture and subsequently subjected to three parallel experimental arms: 2D culture, 3D spheroid culture, and in vivo subcutaneous tumorigenesis assays (Figure 3B).

In 2D culture, a subset of library-transduced MEFs exhibited markedly enhanced proliferative capacity and formed visible colonies (Figure 3C and Figure S2A). In 3D methylcellulose culture, transduced cells formed spheroids of variable sizes, indicating acquisition of anchorage-independent growth potential [26]. Analysis of sgRNA representation revealed progressive clonal selection during in vitro culture (Figure 3D; Table S4). The functional integrity of the mBRCA-KO library was confirmed by significant negative selection of common essential genes in both 2D and 3D conditions (Figure 3E) [27].

Scatter plot analysis of condition-specific enrichment identified both context-dependent regulators and recurrent tumor suppressor hits across screening conditions (Figure 3F). Critically, *Trp53* and interleukin 17B (*Il17b*) emerged as the most consistently enriched genes across all three conditions, establishing them as predominant rate-limiting dependencies in the context of BRCA-associated gene perturbation. The co-enrichment of a canonical tumor suppressor (*Trp53*) alongside an immunomodulatory cytokine (*Il17b*) suggests that malignant transformation is constrained not only by classical p53-mediated surveillance but also by immune-regulatory mechanisms. While IL-17B is primarily recognized as a member of the IL-17 cytokine family with pro-inflammatory and immunomodulatory functions [37], its recurrent loss-of-function enrichment in our screen suggests a context-dependent role in fibroblast transformation that warrants further mechanistic investigation.

### 2.7. Limited Overlap Between In Vitro and In Vivo mBRCA-KO Screens

Subcutaneous tumors derived from mBRCA-KO-transduced MEFs exhibited strong clonal enrichment of sgRNAs targeting established tumor suppressors, including *Pten*, *Nf1*, and *Trp53*, alongside regulators such as *Il17b* and *Mrgprb1* (Figure 3G and Figure S2B), suggesting that loss of either canonical or context-specific suppressors can independently or cooperatively drive in vivo tumorigenesis [29,38].

Comparative analysis identified 57 enriched genes in 2D culture, 39 in 3D culture, and 14 in subcutaneous tumors (Figure 3H). Only two genes—*Il17b* and *Trp53*—were shared across all three conditions, underscoring that *Trp53* loss represents a universal driver of transformation irrespective of selective context, while the broader genetic landscape of enriched hits is substantially shaped by the nature of the selective environment. These findings indicate that while 2D and 3D in vitro systems capture overlapping biological phenotypes, the in vivo tumor microenvironment imposes distinct and more stringent selective pressures, resulting in a restricted and partially divergent set of driver genes. These findings highlight both the complementary value and inherent limitations of in vitro transformation assays as surrogates for in vivo tumorigenic potential, and argue that in vivo screening remains essential for the identification of *bona fide* drivers of tumor establishment.

### 2.8. Functional Enrichment Highlights Developmental and Endothelial Regulatory Pathways

GO enrichment analysis of genes identified across 2D, 3D, and in vivo mBRCA-KO screens revealed significant enrichment in biological processes, including gland development, the regulation of endothelial cell proliferation, endothelial cell proliferation, the regulation of plasma lipoprotein particle levels, the positive regulation of cholesterol efflux, gliogenesis, and hair cycle-related processes (Figure 3I). Enrichment of endothelial proliferation-related terms suggests that loss of certain BRCA-associated genes may modulate angiogenic or tumor microenvironmental interactions during tumor growth. Concurrently, lipid metabolism-related terms imply potential metabolic adaptations that sustain tumor cell proliferation. The enrichment of developmental processes such as gland development and gliogenesis further suggests reactivation of lineage-associated differentiation programs during malignant transformation.

Collectively, mBRCA-KO screening identifies both shared and context-specific genetic drivers of MEF immortalization and tumorigenesis, with limited overlap between in vitro and in vivo conditions, underscoring the importance of microenvironmental context in shaping the functional gene landscape. These findings parallel those obtained from the mLIHC-KO screen, suggesting that context-dependent selective pressure represents a general feature of functional CRISPR screening across distinct tumor models.

### 2.9. In Vivo hBRCA-KO Screening Identifies Key Drivers of Tumorigenesis and Metastasis in Human Fibroblasts

**Figure 4:**
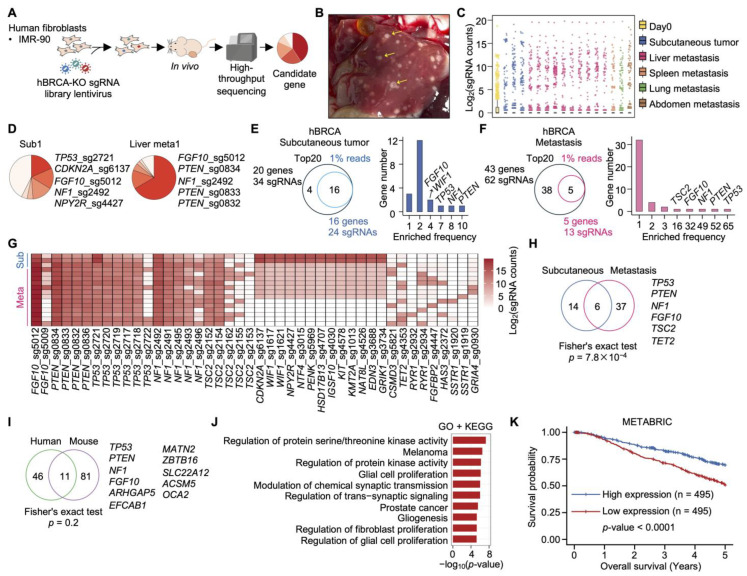
Identification of genes promoting tumorigenesis and metastasis in human fibroblast cell lines using the hBRCA-KO library: (**A**) Schematic overview of the in vivo screening workflow. Human immortalized fibroblast cells were infected with the hBRCA-KO library and injected subcutaneously into mice for tumor formation and metastasis analysis. (**B**) Representative image of liver from mice bearing IMR-90 tumors with metastatic lesions. Yellow arrows indicate liver metastases. (**C**) Boxplot showing the distribution of log_2_-transformed sgRNA counts across the indicated groups. (**D**) Representative pie charts showing the top five enriched sgRNAs in subcutaneous tumor and metastasis from IMR-90 cells. (**E**) **Left**: A Venn diagram showing the overlap of genes represented by the top 20 sgRNAs from each subcutaneous tumor and genes corresponding to sgRNAs occupying > 1% of total reads in the same group. *Right*: Bar plot depicting the occurrence frequency of these genes across subcutaneous tumors, with genes labeled. (**F**) **Left**: A Venn diagram showing the overlap of genes represented by the top 20 sgRNAs from each metastasis and genes corresponding to sgRNAs occupying > 1% of total reads in the same group. *Right*: Bar plot illustrating the occurrence frequency of these genes across metastases, with genes indicated. (**G**) Heatmap of log_2_-transformed sgRNA counts for sgRNAs that appeared in the top 20 sgRNA list of at least two tumors in total across subcutaneous tumors and metastases. (**H**) A Venn diagram showing overlap of genes corresponding to the top 20 sgRNAs in subcutaneous tumors and metastases. (**I**) Comparison of human fibroblast-derived tumorigenesis- and metastasis-related genes with MEF immortalization- and tumorigenesis-related genes. The Venn diagram illustrates the shared genes. (**J**) Heatmap showing GO and KEGG enrichment analysis of genes identified in human fibroblasts as promoting tumorigenesis and metastasis. (**K**) Kaplan–Meier analysis of overall survival in METABRIC breast cancer patients based on tumorigenesis- and metastasis-related genes. Survival stratified by gene expression levels (high expression, top quartile; low expression, bottom quartile). Statistical significance was determined using the log-rank test.

To systematically identify genes promoting tumorigenesis and metastasis in human fibroblasts, we performed in vivo functional screening using the human BRCA-KO (hBRCA-KO) library. Immortalized IMR-90 human fibroblasts were transduced with the pooled hBRCA-KO library and subcutaneously injected into immunocompromised mice for tumor formation and metastatic analysis (Figure 4A and Figure S3A). Multiple metastatic lesions in liver, lung, spleen and abdomen were observed in tumor-bearing mice (Figure 4B), confirming that this model faithfully recapitulates distant metastatic progression.

Deep sequencing of sgRNAs from subcutaneous tumors and matched metastatic lesions revealed substantial inter-sample heterogeneity in sgRNA distributions (Figure 4C; Table S5). While most sgRNAs were present at low abundance, a limited number were markedly enriched within tumors, indicative of strong clonal selection during tumor growth and metastatic dissemination. In samples from a representative mouse, the dominant sgRNAs in the primary subcutaneous tumor targeted *TP53*, *CDKN2A*, *FGF10*, *NF1*, and *NPY2R* (Figure 4D). In contrast, the matched hepatic metastasis was preferentially enriched for sgRNAs targeting *FGF10*, *PTEN* (three independent sgRNAs), and *NF1* (Figure 4D), indicating both shared and metastasis-specific selective pressures between primary and disseminated lesions.

### 2.10. Convergent and Divergent Genetic Determinants of Tumor Growth and Metastasis Colonization

Analysis of clonal enrichment across primary and metastatic lesions revealed distinct yet partially convergent genetic architectures underlying tumor initiation and metastatic progression (Figure 4D–H and Figure S3B). In subcutaneous tumors, clonal dynamics were dominated by a restricted set of recurrently lost genes (*PTEN*, *NF1*, *TP53*, *FGF10*, and *WIF1*), consistent with strong positive selection for inactivation of canonical tumor suppressor and growth-regulatory pathways during primary tumor establishment (Figure 4E). The recurrence of these hits across independent tumor samples argues against stochastic clonal drift and instead implicates these genes as *bona fide* rate-limiting suppressors of tumorigenesis in the context of BRCA-associated genomic instability.

Metastatic lesions exhibited a broader and partially distinct pattern of clonal enrichment, with *TP53*, *PTEN*, *NF1*, *FGF10*, and *TSC2* emerging as the most recurrently identified drivers (Figure 4F). The expanded genetic diversity observed in metastases relative to primary tumors suggests that metastatic colonization imposes additional and partially non-overlapping selective pressures beyond those governing primary tumor growth, potentially reflecting the distinct biological demands of extravasation, survival in the circulation, and establishment at distant sites.

Cross-condition comparison identified six genes shared between primary tumors and metastases: *TP53*, *PTEN*, *NF1*, *FGF10*, *TSC2*, and *TET2* (Figure 4H). This core set of shared drivers likely represents fundamental dependencies for tumor cell survival and proliferative fitness that are maintained across both local and disseminated disease contexts. Notably, *FGF10* loss was among the most consistently enriched events across all conditions—including heatmap analysis demonstrating the enrichment of *FGF10*_sg5012 across all tumors analyzed (Figure 4G)—positioning it as a potentially underappreciated suppressor of BRCA-associated tumor progression whose mechanistic contributions merit further investigation. Collectively, these findings establish that while primary tumor growth and metastatic dissemination share a common core of genetic dependencies, metastasis is associated with a broader and more heterogeneous landscape of cooperating driver events.

### 2.11. Functional Enrichment and Clinical Relevance of Identified Genes

The cross-species conservation of identified drivers was assessed by comparing human tumorigenesis- and metastasis-related genes (57 genes) with those identified in MEF-based mBRCA-KO screening (92 genes). Only 11 genes overlapped between the two species (Figure 4I), and this overlap was not statistically significant, highlighting substantial differences attributable to cellular background and experimental systems.

GO and KEGG enrichment analysis of human tumorigenesis- and metastasis-related genes (Figure 4J) revealed significant enrichment, including the regulation of protein serine/threonine kinase activity, the regulation of protein kinase activity, the regulation of fibroblast proliferation, glial cell proliferation, gliogenesis, the modulation of chemical synaptic transmission, and the regulation of trans-synaptic signaling, as well as cancer-related pathways encompassing melanoma and prostate cancer. Many of these pathways converge on kinase signaling networks, particularly the PI3K/AKT/mTOR and MAPK pathways, which are well-established drivers of breast cancer progression and metastatic dissemination. The clinical relevance of these candidate genes was further evaluated using the METABRIC cohort (Figure 4K). Patients with lower expression of these genes exhibited significantly poorer 5-year overall survival than those with higher expression.

### 2.12. Functional Classification and Cross-Species Network Analysis of LIHC- and BRCA-Derived Diver Genes

To systematically characterize the biological functions of genes identified from both LIHC and BRCA screens, candidate driver genes were categorized into nine functional groups (Table 1): (i) core tumor suppressor pathways & cell cycle regulation, (ii) epigenetic and chromatin regulation, (iii) growth factor and receptor tyrosine kinase signaling, (iv) immune microenvironment modulation, (v) metabolic reprogramming & xenobiotic metabolism, (vi) neuroendocrine and ion channel signaling, (vii) extracellular matrix remodeling & cell adhesion, (viii) hormonal and endocrine regulation, and (ix) transporter & membrane protein. This classification underscores the diverse biological processes contributing to tumor initiation, progression, and metastatic dissemination.

Among the human LIHC and BRCA datasets, a core overlapping gene set was identified comprising *PTEN*, *TP53*, *CDKN2A*, *TSC2*, *NF1*, *CREBBP*, *EGFR*, *C6*, *HSD17B13*, and *ADRA1A*. These shared genes are well-established regulators of tumorigenesis and metastatic dissemination, primarily functioning through control of cell cycle progression, PI3K/AKT/mTOR and MAPK signaling, chromatin remodeling, and growth factor-mediated transcriptional programs. Beyond these canonical oncogenic regulators, the functional inactivation of metabolic enzyme-encoding genes, including cytochrome P450 family members, alcohol dehydrogenases, and regulators of amino acid and lipid metabolism, may promote HCC and breast cancer progression through impaired detoxification and metabolic reprogramming. Loss of phase I detoxification capacity impairs xenobiotic clearance and increases the accumulation of mutagenic metabolites such as acetaldehyde, thereby enhancing genomic instability [39]. Disruption of bile acid and retinoic acid homeostasis may further sustain a pro-inflammatory and pro-fibrotic microenvironment that facilitates liver tumor development [40,41,42]. Meanwhile, inactivation of genes involved in glucose and lipid metabolism supports aerobic glycolysis and lipid accumulation, meeting the energetic and biosynthetic demands of tumor cells [1,32]. Collectively, these alterations create a permissive metabolic environment for tumor initiation and progression. In contrast, cross-species comparison revealed limited overlap with the murine dataset, with only *Trp53*, *Dst*, and *Nf1* consistently identified across human and mouse screens, underscoring substantial context-dependent differences between human and murine fibroblast-based screening systems.

**Figure 5:**
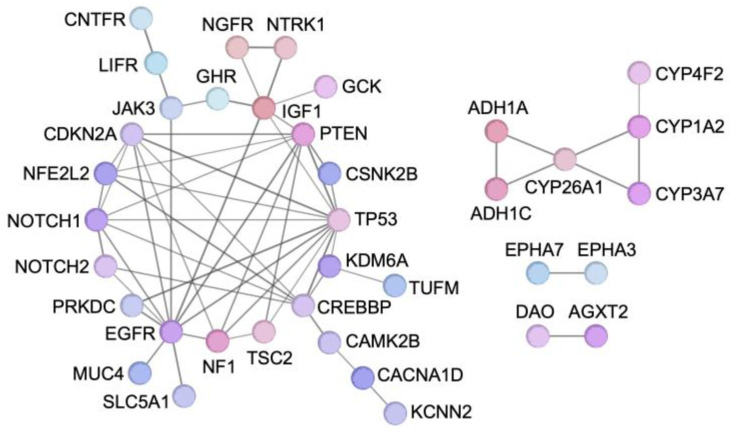
PPI network illustrating the functional interactions among key driver genes of tumorigenesis and metastasis identified through the hLIHC-KO and hBRCA-KO screenings.

Protein–protein interaction (PPI) network analysis revealed two major functional clusters (Figure 5). The first cluster comprised MAOA, ADH1A, ADH1C, CYP26A1, CYP3A7, CYP1A2, and CYP4F2—enzymes responsible for the oxidative metabolism of key endogenous and exogenous molecules, primarily functioning in neurotransmitter degradation (MAOA), alcohol detoxification (ADH1A and ADH1C), and phase I oxidation of drugs, fatty acids, and vitamins (CYP26A1, CYP3A7, CYP1A2, and CYP4F2). This metabolic cluster highlights the potential importance of xenobiotic metabolism and lipid oxidation in shaping the tumor microenvironment and modulating cancer progression.

The second cluster revealed a highly interconnected regulatory network centered on NFE2L2, NOTCH1, NOTCH2, KDM6A, IGF1, EGFR, NTRK1 and NGFR. These genes form a complex interaction network integrating cell cycle regulation, oxidative stress response, epigenetic modification, receptor tyrosine kinase signaling, and neurotrophic pathways. The dense connectivity within this cluster suggests coordinated regulation of proliferative signaling, lineage plasticity, and stress adaptation programs that collectively drive tumor development and metastatic competence.

Collectively, these results demonstrate that LIHC- and BRCA-derived driver genes converge on core tumor suppressor networks, metabolic adaptation pathways, and growth factor-mediated signaling circuits, while also revealing substantial species- and context-specific differences in tumorigenic mechanisms.

## 3. Discussion

In this study, we deployed customized LIHC- and BRCA-focused CRISPR/Cas9 knockout libraries to systematically dissect the functional impact of the genetic perturbations of cancer-associated genes in both murine and human fibroblast systems. By integrating 2D monolayer culture, 3D spheroid assays, and in vivo subcutaneous tumor models, we captured distinct selective pressures governing tumor initiation, clonal evolution, and metastatic dissemination, providing a multi-dimensional framework that extends beyond conventional single-context CRISPR screens.

A central finding across both screens was the predominant and near-universal contribution of *Trp53*/*TP53* loss to fibroblast transformation. *Trp53* was the sole gene consistently enriched across all experimental platforms in the mLIHC-KO screen, reinforcing its fundamental tumor-suppressive role [43,44]. Notably, *Nat2* emerged as a candidate driver of hepatic tumorigenesis, with both functional screening and clinical data supporting a tumor-suppressive role for *NAT2* in LIHC. *Trp53* and *Il17b* were the most consistently enriched genes across all three conditions in the mBRCA-KO screen. The recurrent enrichment of *Il17b* suggests that, beyond canonical p53-dependent tumor suppression, immunomodulatory signaling may also contribute to tumor development in this model. Beyond these core dependencies, additional context-specific hits—including metabolic regulators involved in fatty acid oxidation and xenobiotic metabolism—suggest that metabolic reprogramming constitutes a critical adaptive mechanism during early tumorigenesis, consistent with emerging evidence that metabolic flexibility facilitates both proliferative expansion and stress tolerance [45,46].

In human fibroblast models, hLIHC-KO and hBRCA-KO screens identified gene losses that promoted both primary tumor formation and metastasis. While canonical tumor suppressors such as *NF1* and *TP53* were enriched in both settings, metastases showed additional selection for genes including *PTEN*, *TSC2*, and *TET2*, supporting a clonal evolution model in which metastatic progression requires fitness traits beyond those needed for primary tumor growth. Among the metastasis-specific candidate genes identified in our hLIHC screen, *PRSS8* stands out as a critical suppressor and its downregulation has been shown to fuel metastatic progression by augmenting the invasive potential of tumor cells [47].

The strong clonal dominance observed in several in vivo tumors most likely reflects genuine positive selection rather than stochastic bottleneck effects, as identical dominant sgRNAs recurred across independent tumor samples from different animals. Nevertheless, bottleneck effects during engraftment and background somatic mutations accumulated during in vitro expansion cannot be formally excluded, reinforcing the importance of validating individual candidates through independent functional assays.

The functional categorization and PPI network analysis of LIHC- and BRCA-derived driver genes delineated two major biological clusters: an oxidative metabolic cluster comprising xenobiotic and lipid-metabolizing enzymes, and a densely interconnected regulatory cluster. These genes collectively governed proliferative signaling, lineage plasticity, and stress adaptation. Clinically, mutation or reduced expression of these candidate genes was significantly associated with adverse survival outcomes in LIHC and BRCA patients, supporting their clinical relevance.

Several limitations merit acknowledgment. The fibroblast-based transformation models employed here diverge from the epithelial cellular context of LIHC and BRCA, and gene dependencies identified in fibroblasts may not fully recapitulate those operative in hepatocytes or mammary epithelial cells. Additionally, loss-of-function screening is intrinsically limited to the detection of tumor suppressor-like events. Oncogenic gain-of-function drivers are not captured by this approach and would require complementary screening strategies. Variability in sgRNA efficiency may also introduce false-negative results, and the relatively modest number of in vivo samples limits statistical power for cross-condition comparisons.

In summary, our study comprehensively delineates a genetic landscape that drives tumorigenesis and progression from non-cancerous cells across different species and contexts, as well as deepens understanding on this fundamental question in cancer field.

## 4. Materials and Methods

### 4.1. Construction of CRISPR/Cas9 SgRNA Plasmid Library

The hLIHC-KO library was constructed to target 641 candidate genes potentially involved in liver cancer progression and metastasis. Gene selection was based on the following criteria: (1) genes with mutation frequencies greater than 1% in LIHC cohorts according to the COSMIC database; (2) genes exhibiting mutation rates exceeding 5% in LIHC datasets from cBioPortal; (3) genes significantly downregulated in primary tumors compared with normal tissues (log_2_ fold change < −1.5) or in metastatic tumors relative to primary tumors (log_2_ fold change < −3), as determined using Firehose datasets. In addition, genes markedly decreased in tumor tissues versus normal tissues (log_2_ fold change < −4) were identified based on data from the CNHPP. Mouse orthologs of the selected human genes were subsequently determined, resulting in 649 murine genes for further investigation. For construction of the human library, sgRNAs were obtained from the genome-wide human CRISPR knockout library H3 (Addgene, Watertown, MA, USA, #133914), whereas mouse-specific sgRNAs were newly designed. Both libraries included AAVS1-targeting sgRNAs, non-targeting controls, and sgRNAs targeting essential genes as internal controls. In total, 4145 sgRNAs were incorporated into the hLIHC-KO library and 4394 sgRNAs into the mLIHC-KO library. The design of the BRCA-KO sgRNA library has been described previously [36].

Custom oligonucleotide pools targeting the selected genes were synthesized by Synbio Technologies (Suzhou, China), followed by PCR amplification and cloning into the pLentiCRISPRv2 vector (Addgene, Watertown, MA, USA, #52961) using Gibson Assembly (EasyGeno Assembly Cloning Kit, Tiangen, Beijing, China, #VI201). The assembled plasmids were introduced into electrocompetent Stable *E. coli* cells (New England Biolabs, Ipswich, MA, USA, #C3040) via electroporation and cultured at 30 °C for 16–20 h to ensure adequate library coverage. Endotoxin-free plasmid DNA was subsequently isolated using the Endo-Free Maxi-Prep Kit (TIANGEN, Beijing, China, #4992194). The complexity and uniform distribution of sgRNAs within the plasmid libraries were evaluated by next-generation sequencing to confirm library quality.

### 4.2. Cell Culture

The human fibroblast cell lines IMR-90 (#SCSP-5013) and HFF-1 (#SCSP-109) were purchased from the Cell Bank of Shanghai Institute of Biochemistry and Cell Biology, Chinese Academy of Sciences (Shanghai, China). HEK293FT cells (#CRL-1573) were obtained from the American Type Culture Collection (ATCC, Manassas, VA, USA). MEFs were isolated from embryonic day 12.5 (E12.5) wild-type BALB/c mouse embryos, enzymatically dissociated using 0.25% trypsin–EDTA, and subsequently plated onto dishes for expansion. All cell lines were maintained in Dulbecco’s Modified Eagle Medium (DMEM; BI, Beit HaEmek, Israel, #06-1055-57-1ACS) supplemented with 10% fetal bovine serum (ExCell, Shanghai, China, #FSP500) and 1% penicillin–streptomycin (VivaCell Biosciences, Shanghai, China, #C3420-0100). Cells were maintained at 37 °C in a humidified incubator with 5% CO2 and passaged using TrypLE (Gibco, Grand Island, NY, USA, #12563029).

### 4.3. Three-Dimensional Spheroid Culture

A total of 5 × 10^6^ MEFs transduced with the sgRNA library were seeded into 90 mm Petri dishes for suspension culture (Biosharp, Beijing, China, #BS-90-D) and cultured in 15 mL of medium containing 0.75% methylcellulose (Sigma, St. Louis, MO, USA, #M0512). The medium was refreshed every 3–4 days. For spheroid passaging, spheroids together with the culture medium were collected, diluted with PBS, and centrifuged at 800 rpm for 15 min. The cell pellet was then resuspended in fresh medium for continued culture.

### 4.4. Lentivirus Production and Infection

Lentiviral particles were generated in HEK293FT cells by co-transfecting the lentiviral vector together with the packaging plasmids pCMVR8.74 and pMD2.G using the Lipofectamine™ 2000 reagent (Thermo Fisher, Waltham, MA, USA, #11668019). Six to twelve hours after transfection, the culture medium was replaced. Viral supernatants were harvested 48–72 h post-transfection and stored at –80 °C until use. Target cells were plated in dishes and exposed to the lentivirus at the desired multiplicities of infection (MOI). Two days after infection, the medium was refreshed, and cells were allowed to expand for an additional five days. After seven days post-infection, cells were harvested as the Day 0 sample, while the remaining cells were maintained for either in vitro cell immortalization and expansion or in vivo tumor models.

### 4.5. Mice

Six- to eight-week-old BALB/c nude mice and wild-type BALB/c mice were purchased from Beijing HFK Bioscience Co., Ltd. (Beijing, China). Animals were maintained under specific pathogen-free conditions in individually ventilated cages with a 12 h light/dark cycle, controlled temperature (25 °C), and relative humidity of 40–60%.

### 4.6. In Vivo Tumor Model

Three to five million sgRNA library-transduced cells were harvested, washed with cold PBS, and resuspended in a 1:1 mixture of PBS (BI, Beit HaEmek, Israel, #02-024-1ACS) and Matrigel (Corning, Corning, NY, USA, #356234). The cell suspension was subcutaneously injected into both flanks of BALB/c nude mice (3–5 mice per group). Tumor growth was monitored weekly using calipers, and tumor volume was calculated according to the formula: tumor volume = 0.5 × length × width^2^. Body weight was measured weekly. At the experimental endpoint (1~2 months), mice were euthanized, and subcutaneous tumors as well as metastatic lesions were harvested for further analysis.

### 4.7. Genomic DNA Extraction

For subcutaneous tumors and metastatic lesions, tissues were processed by mechanical dissociation to obtain single-cell suspensions and then centrifuged for harvesting cell pellets. Cell pellets were lysed with lysis buffer (300 mM NaCl, 0.2% SDS, 2 mM EDTA, 10 mM Tris-HCl, pH 8.0). RNase A (10 mg/mL) was added and incubated at 65 °C for 1 h, followed by proteinase K digestion (10 mg/mL) at 55 °C overnight. Samples were mixed with an equivalent volume of phenol/chloroform/isoamyl alcohol (25:24:1) and centrifuged to separate phases. The aqueous fraction was transferred to a new tube, and DNA was precipitated with isopropanol. Pellets were recovered by centrifugation, washed twice with 80% ethanol, air-dried, and dissolved in nuclease-free water. DNA yield was quantified before downstream analysis.

### 4.8. NGS Library Construction

Genomic DNA was isolated from each screening sample, and sgRNA cassettes were enriched through two successive PCR reactions according to established protocols [48]. The resulting amplicons were purified by gel extraction and prepared as sequencing libraries, which were subsequently analyzed on an Illumina instrument (Novogene Co., Ltd., Beijing, China) using paired-end 150 bp reads.

### 4.9. CRISPR Screen Data Analysis

All sequencing datasets were processed using the MAGeCK software package (v0.5.9.4) [27]. Raw sequencing reads in FASTQ format were mapped to the indicated sgRNA library, and guide-level abundance tables were generated via the count module under default settings, producing matrices for downstream evaluation. For in vitro experiments, gene-level effects were estimated using the mle module. Read counts were normalized with the Day 0 sample or plasmid library as the reference condition to calculate β-scores for each targeted gene.

For subcutaneous tumor and metastatic samples, genes corresponding to the top 20 sgRNAs were selected for downstream analysis. In 2D and 3D culture screens, genes with a β-score greater than 2 were defined as functional gene hits. Genes hits derived from in vivo or in vitro experiments were used for GO and KEGG enrichment analysis, which was performed using ClusterProfiler (v4.12.6). Terms with *p*-values < 0.05 were considered statistically significant.

### 4.10. Survival Analysis

Clinical information for the TCGA-LIHC cohort was retrieved from the GDAC repository via the GDC portal (https://portal.gdc.cancer.gov/, accessed on 7 February 2026), while METABRIC clinical datasets were accessed through cBioPortal (https://www.cbioportal.org/, accessed on 7 February 2026). Patients were grouped according to mutation profiles or the expression patterns of specified gene signatures. Overall survival probabilities were estimated using Kaplan–Meier analysis implemented in the survminer package (v0.5.1) in R (v4.4.1), and statistical significance was assessed with the log-rank test. Overall survival analysis of liver cancer patients based on *NAT2* expression levels was performed using the Kaplan–Meier Plotter online tool (https://kmplot.com/analysis/, accessed on 22 March 2026). Patients were stratified into high- and low-expression groups according to *NAT2* mRNA expression levels, and survival curves were compared using the log-rank test. A *p*-value < 0.05 was considered statistically significant.

### 4.11. Gene Expression Analysis

The expression level of *NAT2* in liver LIHC and normal liver tissues was analyzed using the Gene Expression Profiling Interactive Analysis (GEPIA) web server (http://gepia.cancer-pku.cn/, accessed on 22 March 2026) [49]. Gene expression data were obtained from The Cancer Genome Atlas (TCGA) and the Genotype-Tissue Expression (GTEx) database. Gene expression values were normalized and presented as log_2_(TPM+1), where TPM denotes transcripts per million. Differential expression between tumor and normal samples was assessed using a one-way ANOVA model with a significance threshold of *p* < 0.05. Results were visualized as boxplots generated through the GEPIA platform.

### 4.12. Statistical Analyses

All statistical analyses were conducted in R. Overlap significance between gene sets was evaluated using Fisher’s exact test implemented in the GeneOverlap package. Group comparisons were performed using a two-sided Wilcoxon rank-sum test from the base stats package. Statistical significance was defined as follows: * *p*-value < 0.05, ** *p*-value < 0.01, and *** *p*-value < 0.001.

### 4.13. PPI Analysis

PPI analysis was performed using the STRING database (v12.0). Interactions with a minimum required confidence score ≥ 0.7 (high confidence) were retained for downstream analysis. Disconnected nodes (singletons) were excluded from the network. Network visualization and layout optimization were conducted in Cytoscape (v3.10.4) using the yFiles layout algorithms.

## Figures and Tables

**Table 1:** Functional classification of genetic drivers identified through in vitro and in vivo screening with the LIHC-KO and BRCA-KO libraries. Bold genes denote shared hits between LIHC and BRCA libraries.

Biological Category	LIHC-Human	BRCA–Human	LIHC–Mouse	BRCA-Mouse
Core Tumor Suppressor & Cell Cycle Regulation	***TP53***, ***PTEN***, ***CDKN2A***, ***TSC2***, ***NF1***, *PRKDC*	***TP53***, ***PTEN***, ***CDKN2A***, ***TSC2***, ***NF1***, *RB1*	***Trp53***, ***Nf1***, *Cdkn1a*, *Rb1*, *Tsc1*, *Atr*	***Trp53***, ***Nf1***, *Pten*, *Apc*, *Stag1*, *Stag2*, *Cbfb*, *Atm*
Epigenetic & Chromatin Regulation	***CREBBP***, *KDM6A*, *NFE2L2*	***CREBBP***, *KMT2A*, *TET2*, *TRRAP*, *ZBTB16*	*Kmt2c*, *Bcor*, *Runx1t1*, *Grhl2*, *Fosb*	*Asxl1*, *Ctcf*, *Kat6b*, *Zbtb16*, *Bcorl1*, *Dido1*, *Tbx3*
Growth Factor & Receptor Tyrosine Kinase Signaling	***EGFR***, ***ADRA1A***, *IGF1*, *GHR*, *LIFR*, *CNTFR*, *NTRK1*, *EPHA3*, *EPHA7*, *NOTCH1*, *NOTCH2*, *JAK3*, *CAMK2B*	***EGFR***, ***ADRA1A***, *FGF10*, *WIF1*, *KIT*, *NTRK3*, *ADAMTS5*, *ADAMTS7*, *ARHGAP5*, *EDN3*, *FGFBP2*	*Hgf*, *Erbb4*, *Rps6ka3*, *Adgra1*, *Adgrb3*, *Hhip*	*Fgf10*, *Flt4*, *Ngfr*, *Arhgap5*, *Alx4*, *Rxrg*, *Klb*, *Reln*
Immune Microenvironment Modulation	***C6***, *CXCL2*, *CARD11*, *FPR2*, *FCN3*, *RNASE2*, *SELP*, *CFTR*	***C6***, *CCL28*, *CASC5*, *CTSG*	*Ccl21c*, *Hamp*, *Cxcl14*, *Il13ra2*, *Il1rl1*, *Cfhr1*, *Cd209g*, *Ceacam1*, *Defa2*	*Il17b*, *Il33*, *Btnl9*, *Cd300lg*, *C1qtnf7*, *Clec4g*, *Cmtm5*
Metabolic Reprogramming & Xenobiotic Metabolism	*CYP1A2*, *CYP3A7*, *CYP3A43*, *CYP26A1*, *CYP4F2*, *CYP8B1*, *ADH1A*, *ADH1C*, *DAO*, *GCK*, *KMO*, *AGXT2*, *ACSM2B*	*MAOA*, *ACACB*, *ACSM5*, ***HSD17B13***, *NAT8L*	*Cyp1a1*, *Cyp2j8*, *Cyp2j11*, *Cyp4a32*, *Cyp4f40*, *Adh4*, *Gls2*, *Acmsd*, *Nat2*, *Bhmt*, *Eno3*, *Nr1i2*	*Aldh1l1*, *Acsm5*, *Adipoq*, *Pck1*, *Glyat*, *Plin1*, *Saa4*, *Afp*, *Klb*, *Ces1d*, *Ces1g*
Neuroendocrine, Synaptic & Ion Channel Signaling	*NGFR*, *GABRP*, *KCNN2*, *KCND3*, *CACNA1D*, *CHRM2*, *PVALB*, *TENM3*, *ADRA1A*	*NTRK3*, *GRIA4*, *GRIK1*, *NPY2R*, *SSTR1*, *PENK*, *RYR1*, *SCN3A*, *NTF4*, *ADRA1A*	*Pvalb*, *Oxt*, *Lrrn1*, *Ttr*, *Prima1*, *Fxyd1*, *Ppp1r1a*	*Ngfr*, *Cacna1e*, *Gpr12*, *Gpr17*, *Mrgprb1*, *Slc22a12*
Extracellular Matrix Remodeling & Cell Adhesion	*MUC4*, *SPON1*, *KANK4*, *MACF1*, *ARHGEF10*, *CHST4*, *LRRC55*, *FAM151A*	*FAT2*, *COL6A6*, *MATN2*, *CSMD3*, *ASTN1*, *BMPER*, *MAMDC2*, *IGSF10*, *HAS3*	***Dst***, *Vcan*, *Muc6*, *Fbn2*, *Ccbe1*, *Mep1b*, *Mug2*	***Dst***, *Emilin3*, *Cadm3*, *Obscn*, *Neb*, *Dnah8*, *Dnah9*, *Fmn2*, *Gfap*, *Matn2*, *Krt14*, *Csmd1*, *Klhl29*, *Klhl31*
Hormonal & Endocrine Regulation	***HSD17B13***, ***ADRA1A***, *ADGRG7*, *GHR*	***HSD17B13***, ***ADRA1A***, *AR*, *ADRB2*, *EDN3*	*Hsd17b6*, *Shbg*, *Thrsp*, *Angptl6*, *Tctex1d1*	*Adipoq*, *Rxrg*, *Plin1*, *Scara5*, *Dct*
Transporter & Membrane Protein	*SLC22A1*, *SLC5A1*, *ABCA8*, *CFTR*, *ATP13A4*, *CSNK2B*	*SLC22A12*	*Slco4c1*, *Npc1l1*, *Mfsd2a*, *Tmprss6*	*Slc27a6*, *Slc22a12*, *Abca8b*, *Abcb5*, *Npc1l1*
Uncharacterized/Structural/Others	*GYG2*, *PRSS8*, *C6orf120*, *KIAA1109*, *TUFM*, *EIF6*, *MME*, *WNK2*, *SERPINA11*, *LRRN1*, *CCDC64B*, *CTSF*	*EFCAB1*, *FAM135B*, *HNRNPK*, *NWD2*, *OCA2*, *PTCHD3*	*Ay761184*, *Gm10591*, *Gm7849*, *Ttc36*, *Iglon5*, *Klk10*	*1700009n14rik*, *Gm6614*, *Capn11*, *Efcab1*, *F10*, *Hepacam*, *Hs3st4*, *Pamr1*, *Oca2*, *Nbas*, *Ropn1*, *Samd5*, *Tfip11*, *Tnmd*, *Trip11*, *Gpihbp1*, *Gpld1*, *Lrrc2*, *Pld5*, *Fhl1*, *Hspb7*, *Cyc1*, *Dlk1*, *Aoc3*, *Aspa*, *Nts*, *Csn3*

## Data Availability

The datasets presented in this study can be found in SRA: PRJNA1426757. All other data supporting the findings of this study are available within the article or Supplementary Material.

## Supplementary Materials

The following supporting information can be downloaded at https://www.mdpi.com/article/10.3390/ijms27073223/s1.

## Abbreviations

The following abbreviations are used in this manuscript:

LIHC	Liver Hepatocellular Carcinoma
BRCA	Breast Carcinoma
MEFs	Mouse Embryonic Fibroblasts
2D	Two-Dimensional
3D	Three-Dimensional
RB	Retinoblastoma
hTERT	Human Telomerase Reverse Transcriptase
TCGA	The Cancer Genome Atlas
KO	Knockout
hLIHC-KO	Human LIHC-specific KO
CNHPP	Chinese National Human Proteome Project
mLIHC-KO	Mouse LIHC-specific KO
IQR	Interquartile Range
MOI	Multiplicities of Infection
GO	Gene Ontology
WT	Wild Type
KEGG	Kyoto Encyclopedia of Genes and Genomes
BRCA-KO	BRCA-specific KO
mBRCA-KO	Mouse BRCA-KO
hBRCA-KO	Human BRCA-KO
PPI	Protein–Protein Interaction
