# Supplementary material for: Functional CRISPR Screens Define Genetic Drivers for Cancer Transformation and Progression from Non-Cancerous Cells
Source: Int J Mol Sci. 2026 Apr 2;27(7):3223. doi: 10.3390/ijms27073223 (PMC13072818; doi:10.3390/ijms27073223)
Supplement: Supplementary file 1 [file ijms-27-03223-s001.zip › Supplementary Materials.pdf]

Supplementary Materials

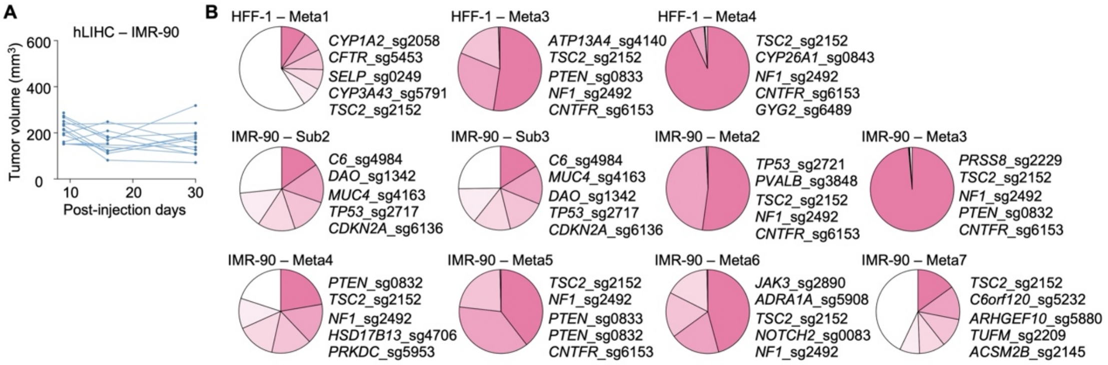

Supplementary Figure S1. *In vivo* tumor growth and sgRNA enrichment profiles in IMR-90 and HFF-1–derived tumors. (**A**) Tumor growth curves representing the size of subcutaneous tumors formed by IMR-90 cells in mice over time. (**B**) Pie charts showing the top five enriched sgRNAs in each tumor. Groups include subcutaneous tumors and liver metastases from IMR-90 and HFF-1 cells.

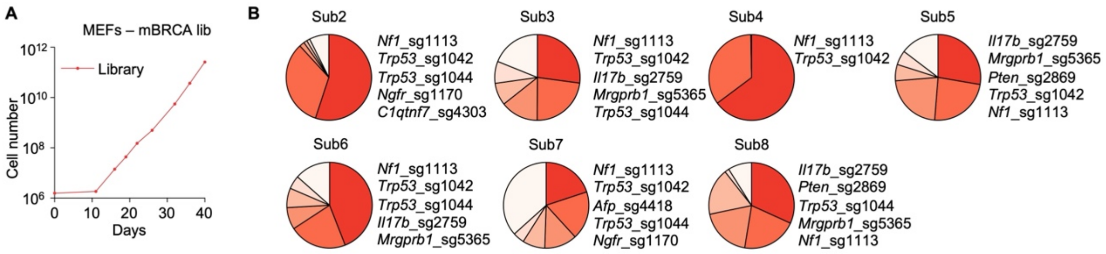

Supplementary Figure S2. *In vivo* and *in vitro* screening of MEFs using the mBRCA-KO library. (**A**) Cell growth assay quantifying cell numbers during 2D screening after infection with the mBRCA-KO library. (**B**) Pie charts showing the top five enriched sgRNAs in each tumor.

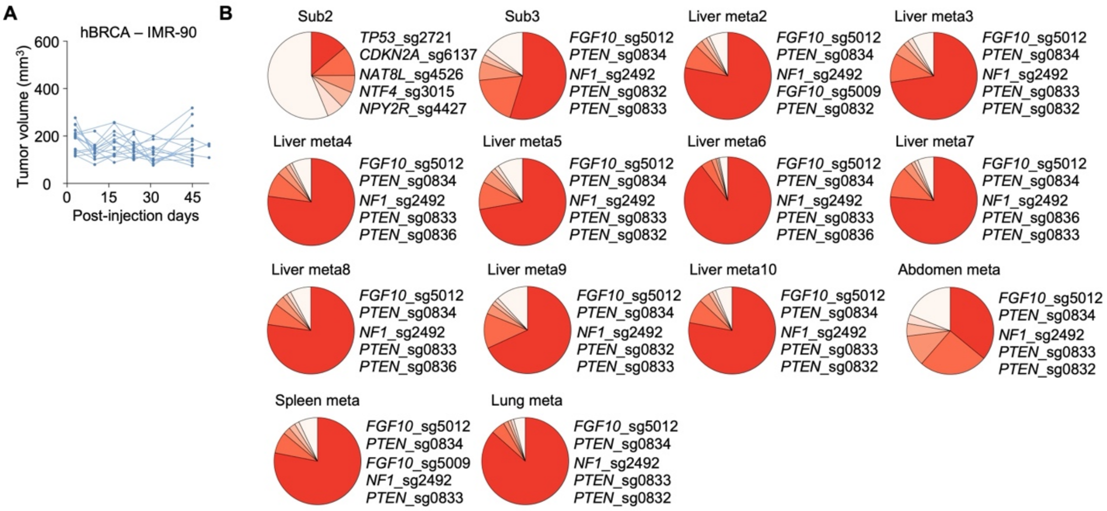

Supplementary Figure S3. *In vivo* screening of human fibroblast cell lines using the hBRCA-KO library. (**A**) Tumor growth curves representing the size of subcutaneous tumors formed by IMR-90 cells in mice over time. (**B**) Pie charts showing the top five enriched sgRNAs in each subcutaneous tumor and metastasis from IMR-90 cells.
